# Duodenal Stenosis Complicated by Foreign Body Impaction in a Child With Down Syndrome: A Case Report

**DOI:** 10.7759/cureus.85358

**Published:** 2025-06-04

**Authors:** Rawan Alhalabi, Muhammad Eyad Ba'Ath, Hesham Sayed Ahmad, Mohammed Samer Baki

**Affiliations:** 1 Pediatrics, American Hospital Dubai, Dubai, ARE; 2 Pediatric Surgery, University of Sharjah, Sharjah, ARE; 3 Pediatric Surgery, Buraidah Maternity and Children's Hospital, Buraidah, SAU

**Keywords:** acute abdomen, child, down's syndrome, down syndrome, duodenal stenosis, gastrointestinal obstruction

## Abstract

Down syndrome (DS) is frequently associated with gastrointestinal (GI) anomalies, with duodenal atresia or stenosis being among the most common. We report a five-year-old boy with DS and hypothyroidism who presented with an acute abdomen and was found to have duodenal stenosis complicated by foreign body ingestion. Surgical intervention revealed significant duodenal narrowing with the impaction of 239 date seeds. This case highlights the importance of early screening for GI anomalies in DS patients, especially when symptoms are atypical or prolonged.

## Introduction

Down syndrome (DS), or trisomy 21, is the presence of a third copy of chromosome 21. It represents the most common chromosomal abnormality in humans [1-4]. The incidence ranges from one in 319 to one in 1,000 live births. Individuals with DS exhibit a wide range of clinical features, including distinctive facial features, congenital heart defects (CHDs), gastrointestinal (GI) abnormalities, hypothyroidism, and various neurological impairments [1,2].

Among GI abnormalities, intestinal obstruction is one of the most common structural defects associated with trisomy 21 and is usually diagnosed in infancy or before the age of four years [3-6]. Here, we report a case of a five-year-old male with DS who was diagnosed with duodenal stenosis complicated by foreign body impaction.

## Case presentation

**Figure 1:**
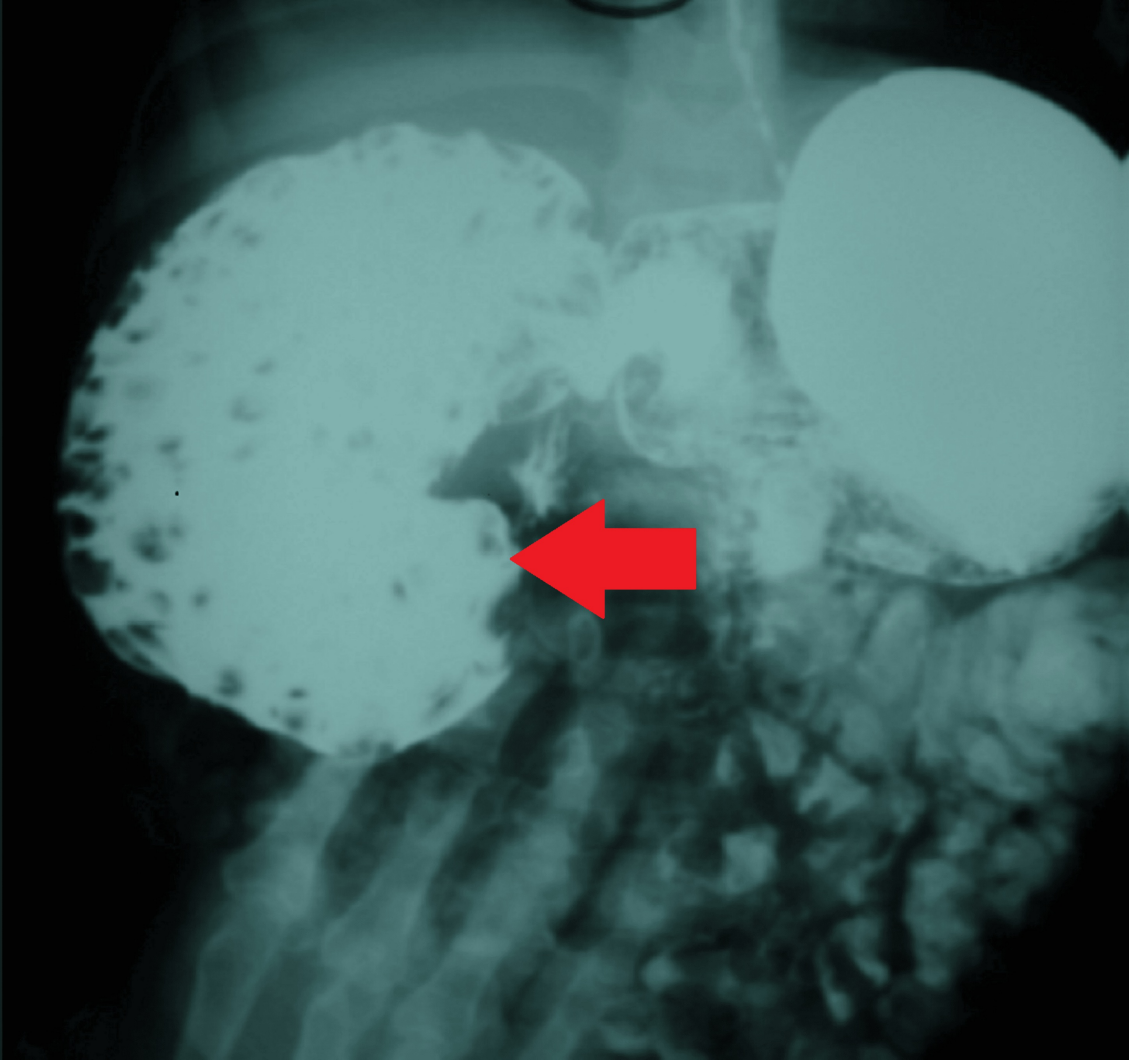
Upper GI barium study Contrast study showing severe dilatation of the second part of the duodenum with numerous filling defects indicative of foreign body impaction (red arrow).

A five-year-old male with a known diagnosis of DS and congenital hypothyroidism presented to the emergency department with a complaint of multiple episodes of bilious vomiting and generalized abdominal pain for one day. No fever or other symptoms were reported. His chronic constipation had previously been attributed to his hypothyroidism, and he had multiple past episodes of fecaloma that were managed conservatively. On examination, his abdomen was soft and distended, with a palpable mass in the right hypochondrium. Abdominal ultrasonography revealed a hyperechoic area in the same region, suspected to be fecal material. A subsequent barium study showed delayed passage of contrast from the duodenum to the jejunum (Figure 1). The duodenum appeared distended and was filled with multiple foreign bodies, suggesting subtotal obstruction.

**Figure 2:**
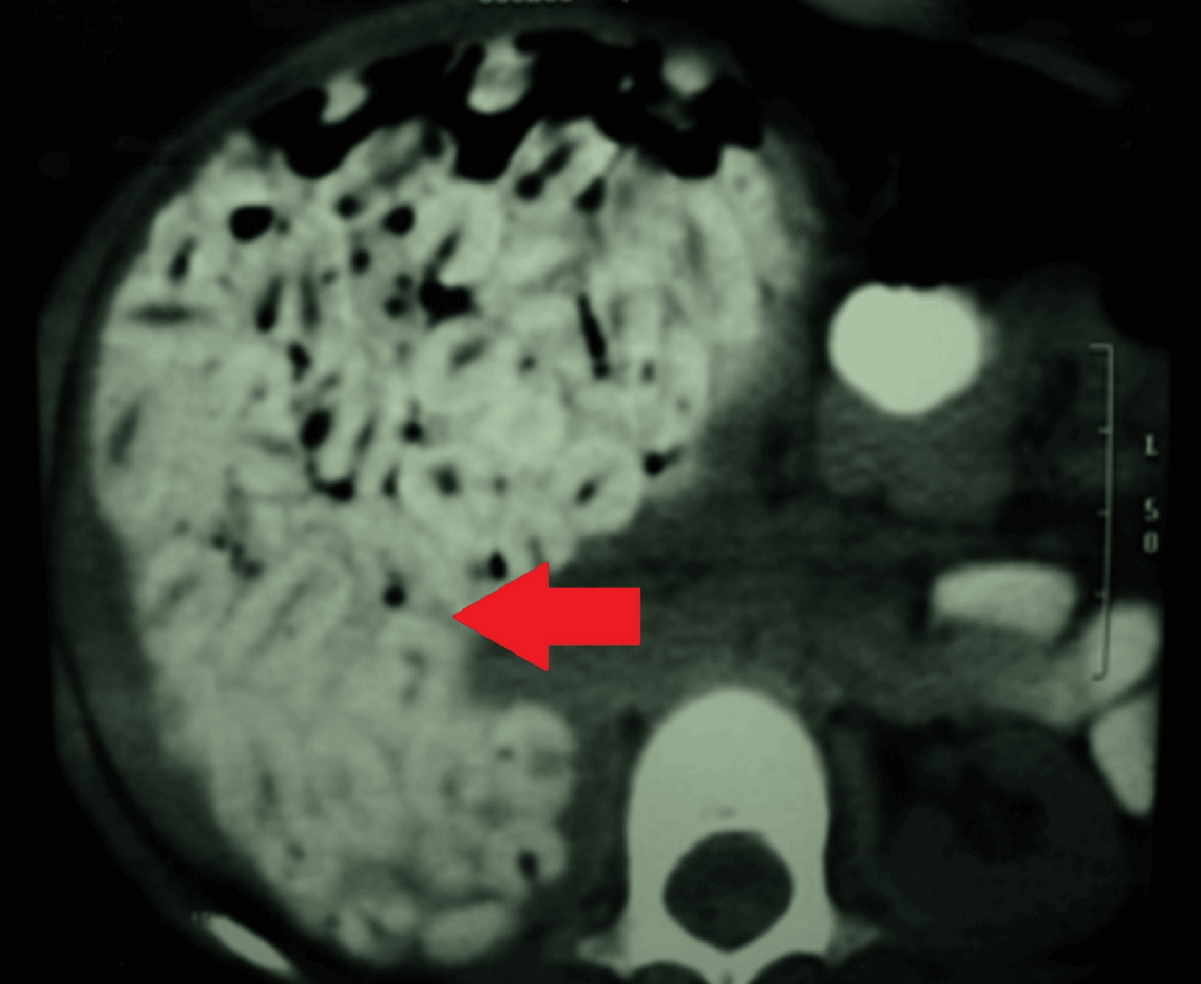
CT-scan abdomen with contrast CT scan with axial view of the dilated duodenum filled with dates’ seeds. Note the distinctive shape of the seed with a central, longitudinal groove (red arrow).

A CT scan confirmed the presence of numerous radiopaque foreign bodies within the stomach and right upper quadrant-intra-intestinal (Figure 2).

**Figure 3:**
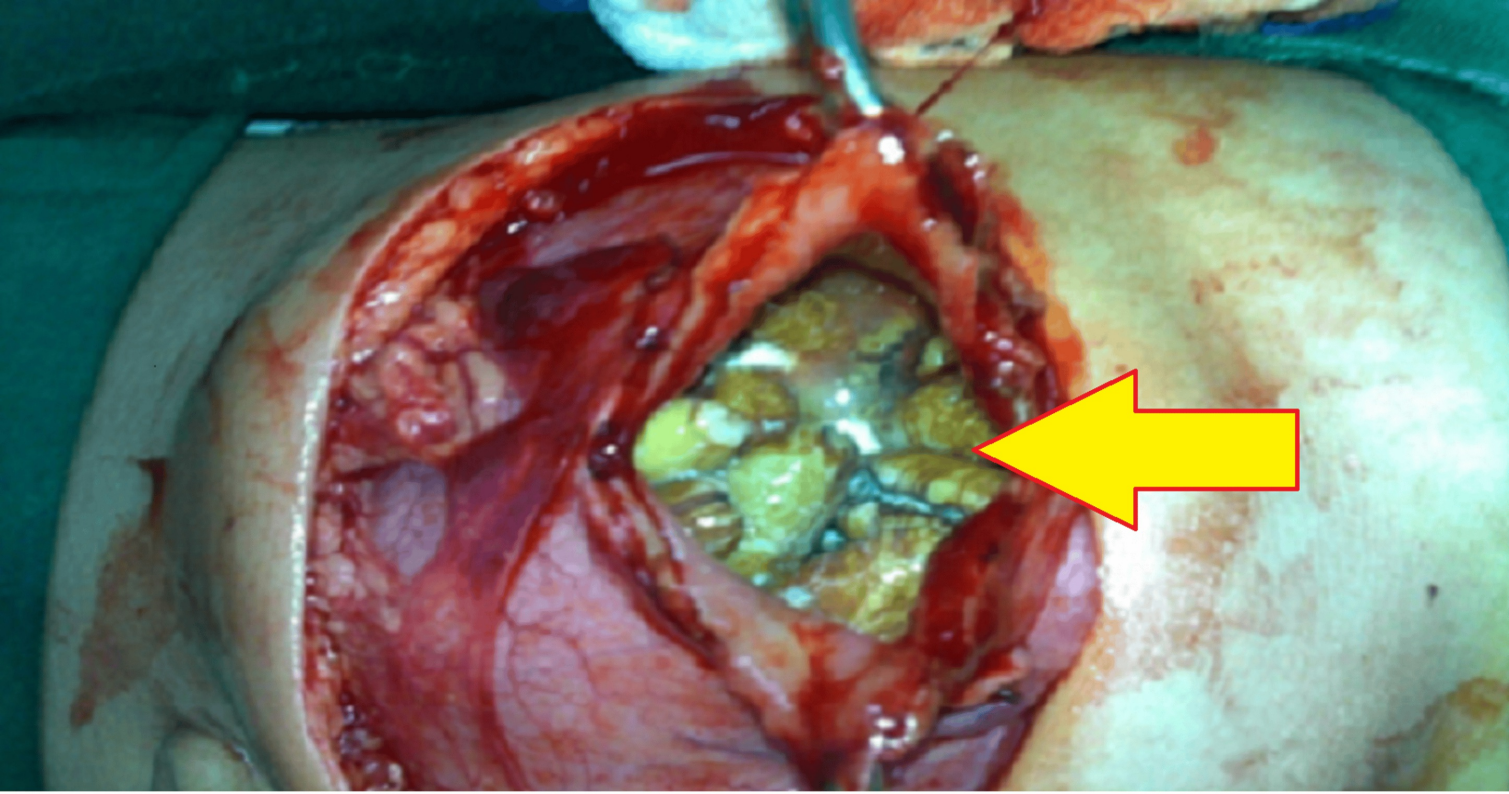
Surgical view Intra-operative view of the impacted foreign bodies (yellow arrow) during duodenotomy.

The patient underwent exploratory laparotomy. He was found to have marked dilatation of the first and second part of the duodenum and a stenosis at the distal second part. A duodenostomy was performed, followed by removal of the foreign bodies and a Roux-en-Y duodenojejunostomy. At least 239 date seeds were retrieved from the duodenum (Figure 3).

The patient tolerated the procedure well, with no postoperative complications noted on follow-up.

## Discussion

GI abnormalities are common in individuals with DS. These include gastroesophageal reflux, intestinal malformations, Hirschsprung disease, chronic constipation or diarrhea, imperforate anus, and celiac disease [1,4]. While CHDs remain the leading cause of mortality and morbidity in infants with DS [1,3], duodenal obstruction is the most frequent congenital intestinal anomaly in this population, with an incidence of 1% to 6.7% [4,5].

Duodenal atresia/stenosis arises from a failure of the duodenal lumen to recanalize during the eighth week of gestation [4]. Classic presentation includes bilious vomiting and abdominal distention, often diagnosed by the typical "double bubble" sign on plain abdominal radiographs [1,4,6]. In cases of partial obstruction (stenosis), symptoms may be subtle or absent, especially in patients with developmental delay, which can lead to delayed diagnosis [6,7]. Of note, annular pancreas is frequently associated with duodenal obstruction in DS and is now considered a consequence rather than a cause of the anomaly [4,6-10]. Anatomically, duodenal obstructions are classified as either atresias or stenoses. An incomplete obstruction, due to a fenestrated web or diaphragm, is considered a stenosis. Most stenoses involve the third and/or fourth part of the duodenum. Atresias, or complete obstructions, are further classified into three morphologic types. Type I atresias account for >90% of all duodenal obstructions and contain a luminal diaphragm that includes mucosal and submucosal layers. A diaphragm that has ballooned distally (windsock) is a type I atresia. Type II atresias are characterized by a dilated proximal and collapsed distal segment connected by a fibrous cord. Type III atresias have an obvious gap separating the proximal and distal duodenal segments. Duodenal atresia is frequently associated with other congenital anomalies, with approximately 30-50% of cases occurring in patients with trisomy 21 [11].

Historically, mortality among neonates with DS and duodenal anomalies reached up to 55%, mainly due to complications such as pneumonia and CHD [4,6,8]. Nevertheless, outcomes have enhanced noticeably in recent decades, with survival rates rising to 88%, as reported by Wesley and Mahour [10]. Attributed to advanced anesthesia and surgical techniques, perioperative care, early cardiac screening, and enhanced nutritional support [10].

While barium contrast studies can be diagnostically useful in evaluating upper GI tract abnormalities, their use may be limited in cases of suspected bowel obstruction or perforation due to the risk of chemical peritonitis and delayed clearance. In such situations, water-soluble contrast agents are generally preferred. This approach is supported by clinical guidelines, including those of the American College of Radiology [12].

In our case, the chronic constipation secondary to hypothyroidism masked the underlying duodenal stenosis. Lack of chronic, repetitive bilious vomiting confused the clinical picture further, as this would have been expected in high intestinal obstruction and duodenal stenosis [1-5]. Furthermore, individuals with DS are at increased risk for foreign body ingestion due to cognitive and behavioral challenges [7]. It is likely that some of the fecalomas previously treated were wrongly labelled and were, in fact, impacted duodenal foreign bodies, which exacerbated the obstruction and precipitated surgical intervention. This case underscores the importance of early screening for duodenal anomalies in children with DS, especially when other risk factors or unusual presentations are involved.

## Conclusions

Duodenal stenosis may remain undiagnosed for years in children with DS, particularly when other comorbidities overshadow its subtle or intermittent symptoms. This diagnostic delay can contribute to repeated hospital visits, nutritional deficit, and avoidable complications such as foreign body ingestion. Clinicians should maintain a high index of suspicion for congenital GI anomalies in this population when they manifest persistent gastrointestinal symptoms. Early recognition through appropriate imaging and multidisciplinary evaluation can facilitate timely intervention, improve quality of life, and prevent potentially serious outcomes. Based on our experience with this case, we recommend evaluating for intestinal obstruction, whether partial or complete, in children with DS who present with persistent vomiting, abdominal pain, or distention. This should also be considered in intellectually disabled children with a potential history of foreign body ingestion.
